# Requirement of Heterogeneous Nuclear Ribonucleoprotein C for *BRCA* Gene Expression and Homologous Recombination

**DOI:** 10.1371/journal.pone.0061368

**Published:** 2013-04-09

**Authors:** Rachel W. Anantha, Allen L. Alcivar, Jianglin Ma, Hong Cai, Srilatha Simhadri, Jernej Ule, Julian König, Bing Xia

**Affiliations:** 1 Department of Radiation Oncology, The Cancer Institute of New Jersey and Robert Wood Johnson Medical School, New Brunswick, New Jersey, United States of America; 2 MRC Laboratory of Molecular Biology, Cambridge, United Kingdom; Harvard School of Public Health, United States of America

## Abstract

**Background:**

Heterogeneous nuclear ribonucleoprotein C1/C2 (hnRNP C) is a core component of 40S ribonucleoprotein particles that bind pre-mRNAs and influence their processing, stability and export. Breast cancer tumor suppressors BRCA1, BRCA2 and PALB2 form a complex and play key roles in homologous recombination (HR), DNA double strand break (DSB) repair and cell cycle regulation following DNA damage.

**Methods:**

PALB2 nucleoprotein complexes were isolated using tandem affinity purification from nuclease-solubilized nuclear fraction. Immunofluorescence was used for localization studies of proteins. siRNA-mediated gene silencing and flow cytometry were used for studying DNA repair efficiency and cell cycle distribution/checkpoints. The effect of hnRNP C on mRNA abundance was assayed using quantitative reverse transcriptase PCR.

**Results and Significance:**

We identified hnRNP C as a component of a nucleoprotein complex containing breast cancer suppressor proteins PALB2, BRCA2 and BRCA1. Notably, other components of the 40S ribonucleoprotein particle were not present in the complex. hnRNP C was found to undergo significant changes of sub-nuclear localization after ionizing radiation (IR) and to partially localize to DNA damage sites. Depletion of hnRNP C substantially altered the normal balance of repair mechanisms following DSB induction, reducing HR usage in particular, and impaired S phase progression after IR. Moreover, loss of hnRNP C strongly reduced the abundance of key HR proteins BRCA1, BRCA2, RAD51 and BRIP1, which can be attributed, at least in part, to the downregulation of their mRNAs due to aberrant splicing. Our results establish hnRNP C as a key regulator of *BRCA* gene expression and HR-based DNA repair. They also suggest the existence of an RNA regulatory program at sites of DNA damage, which involves a unique function of hnRNP C that is independent of the 40S ribonucleoprotein particles and most other hnRNP proteins.

## Introduction

hnRNP C is one of the most abundant proteins in the nucleus (∼10 µM). Its two isoforms, hnRNP C1 and C2, form a (C1)_3_C2 tetramer and serve to nucleate the formation of the 40S hnRNP particles, which also contain hnRNP A1, B2, A2 and B1 [1], [2]. The 40S hnRNP particles assemble on nascent transcripts (pre-mRNAs) and are thought to influence their splicing, transport, stability and possibly other aspects of their metabolism. Conflicting reports exist on the sequence specificity and mode of hnRNP C binding to RNA [3]–[6], and how the protein functions remains incompletely understood. Recently, using individual-nucleotide resolution UV cross-linking and immunoprecipitation (iCLIP), it was shown that hnRNP C binds tracts of 4 or more uridines with defined spacing of 165 or 300 nucleotides and, depending on the exact binding locations, can promote either exclusion or inclusion of alternative exons [7]. Moreover, a new study found that hnRNP C directly competes with the splicing factor U2AF65 at splice sites to prevent exonization of *Alu* elements in introns [8]. hnRNP C is essential for mouse development as homozygous mutant embryos are not viable and are resorbed by 10.5 days of gestation [9]. hnRNP C is, however, dispensable for cellular viability, as homozygous null cells were able to grow and differentiate *in vitro* albeit with slower growth than wild type cells [9].

DNA double strand breaks (DSBs) occur due to both endogenous damage and exogenous genotoxic insults. Homologous recombination (HR) and non-homologous end joining (NHEJ) are two main modes of double strand break repair (DSBR) [10]. HR is a largely error-free mechanism that operates primarily during the S/G2 phases of the cell cycle, while the more error-prone NHEJ is the major DSBR mechanism during the G1 phase. Interestingly, the two major breast cancer suppressor proteins BRCA1 and BRCA2 both play critical roles in HR [11], [12]. By affinity purification of endogenous BRCA2, we previously identified a major BRCA2-binding protein, PALB2, which is critical for BRCA2's chromatin association and function in HR-DSBR [13]. Like BRCA2, PALB2 itself is also mutated in breast cancer, pancreatic cancer, ovarian cancer and Fanconi anemia (FA) [14]–[16]. More recently, we and others demonstrated that PALB2 also directly interacts with BRCA1 and connects BRCA1 and BRCA2 in the HR process [17]–[19].

HR is an extremely complex and highly regulated process. The initiation of HR requires the processing of DSBs to generate a long single-stranded DNA (ssDNA) overhang, a step termed resection, and the binding of the ssDNA by the recombination enzyme RAD51 to form a nucleoprotein filament capable of searching for and invading a homologous template [11]. Current evidence suggests that BRCA1 may facilitate HR via at least two mechanisms. First, it appears to promote end resection through its interaction with the resection-capable nucleases MRE11-RAD50-NBS1 (MRN) complex and CtIP [20]–[22]. This may also involve a competitive prevention of the resection-inhibitory effect of 53BP1, a factor that promotes NHEJ [23]. Second, BRCA1 may recruit the PALB2/BRCA2 complex to resected DSBs via its direct interaction with PALB2 [18], [19]. The role of BRCA2 in HR has been extensively studied, and the protein is now believed to function as an essential “mediator” in mammalian cells to promote the loading of RAD51 onto ssDNA and the stability of RAD51-ssDNA nucleoprotein filament during the initial strand invasion step of HR [24]. PALB2 plays a critical role in HR by enabling BRCA2 (and therefore RAD51) recruitment to the chromatin and DSBs [13], [17], [25]. Additionally, PALB2 can interact with RAD51 directly and may be able to stimulate RAD51 loading and activity independent of BRCA2 [26], [27].

Tandem affinity purifications of epitope-tagged PALB2 has led to the identification of BRCA1 and MRG15 as additional components of the PALB2/BRCA2 complex [17], [18], [28], [29]. These findings have significantly advanced our understanding of the regulation of PALB2 and BRCA2 in HR and DSBR. However, the above purifications were all performed using whole cell or nuclear extracts in which the binding between PALB2 and its chromatin-bound partner proteins might have been missed or altered. In this study, we attempted to purify PALB2 from nuclease-solubilized chromatin fractions and identified hnRNP C as a component of PALB2 nucleoprotein complexes. Our results demonstrated that hnRNP C plays a critical role in HR-DSBR and in the regulation of an important set of DNA repair proteins including BRCA1, BRCA2, RAD51 and BRIP1.

## Results

### Presence of hnRNP C in the PALB2-nucleic acid complexes

**Figure 1:**
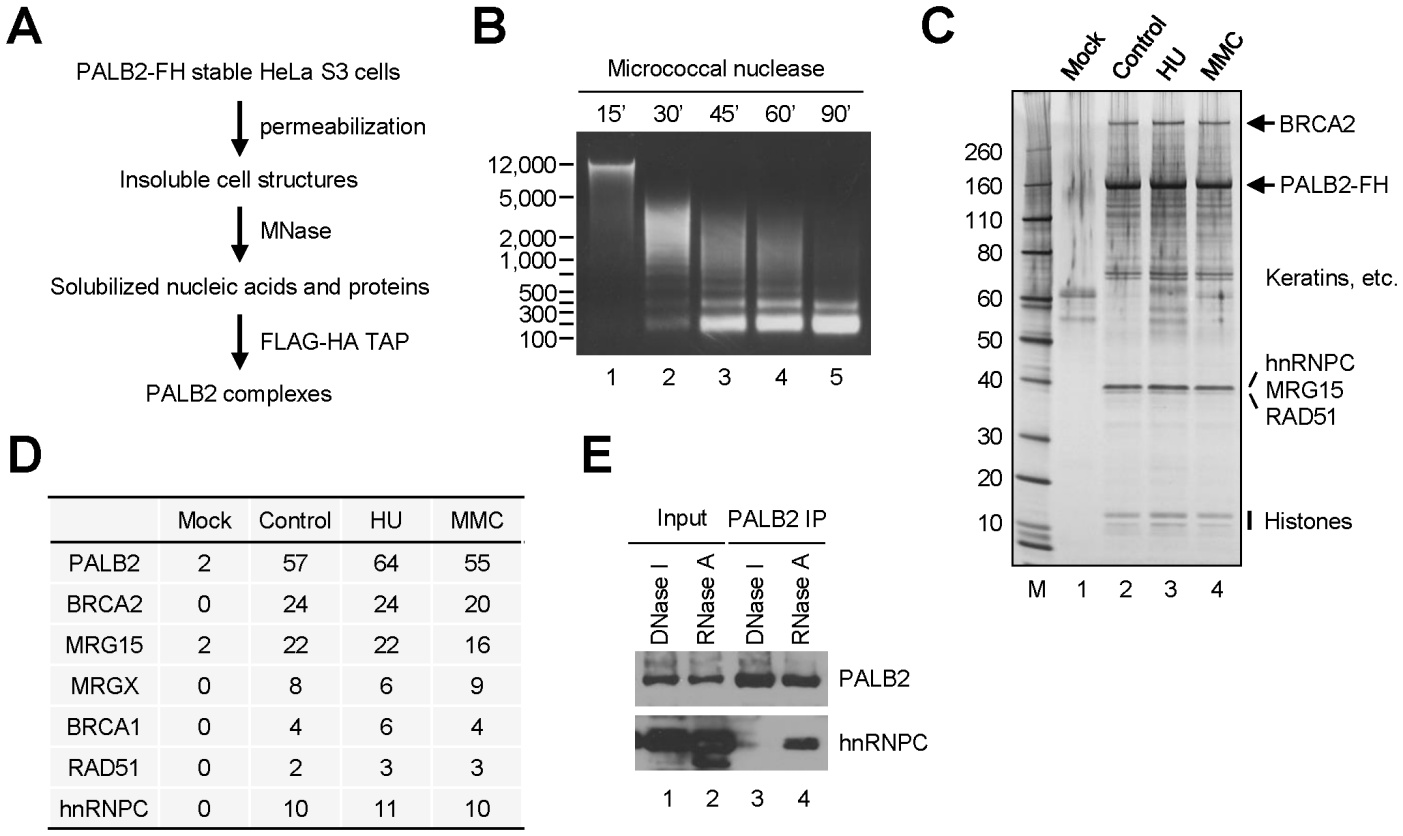
Presence of hnRNP C in PALB2-containing nucleoprotein complexes. **A.** Schematic diagram of the PALB2 purification procedure. **B.** Sizes of DNA fragments in solubilized chromatin fractions after digestion of insoluble nuclear structures with micrococcal nuclease (MNase). **C.** Silver-stained gel showing the components of TAP-purified PALB2 complexes from the solubilized chromatin fraction. **D.** Protein components of the PALB2 complexes identified by liquid chromatography tandem mass spectrometry (LC-MS/MS). The numbers shown are the averages of the numbers of unique peptides detected for each protein in two independent experiments. **E**. The interaction between hnRNP C and PALB2 is mediated by RNA. Nuclear pellets of U2OS cells were digested with DNase I or RNase A, and the nuclease-released components were IPed with a PALB2 antibody. The nuclease-released materials and IPed proteins were analyzed by Western blotting.

To identify proteins that interact with PALB2 in chromatin, we purified FLAG-HA-double tagged PALB2 from micrococcal nuclease (MNase)-solubilized nuclear fractions of HeLa S3 cells stably expressing the tagged protein. As show in Fig. 1A, cells were first permeabilized with a buffer containing low salt and detergent to remove soluble components, the insoluble materials were then treated with MNase to solubilize chromatin DNA and bound proteins, and finally the tagged PALB2 was isolated through tandem affinity purification (TAP). A time course was carried out in order to achieve maximum conversion of insoluble chromatin to the soluble form. We found that incubation of nuclear pellet with MNase for 90 min resulted in nearly complete conversion of genomic DNA to nucleosome-length fragments (∼150 bp) (Fig 1B). Tandem affinity purification of the tagged PALB2 from such maximally solubilized chromatin fraction followed by mass spectrometry analysis identified most known PALB2 binding partners, e.g. BRCA1, BRCA2, RAD51 and MRG15 (Fig. 1C–D). However, there were no significant changes in the amounts of these binding partners in the complexes purified after DNA damage induced by hydroxyurea (HU) and mitomycin C (MMC). As expected, several histones were found in the complexes, but the number of peptides was small and inconsistent for each histone (not shown), likely due to their small size and highly positive charge which may limit the detection by mass spectrometry.

Unexpectedly, hnRNP C was found to be a relatively abundant component of the complexes, indicating that it may either directly interact with PALB2 or its above-noted partner proteins, or reside on the same nucleosomes with PALB2. Another possibility is that hnRNP C and PALB2 may exist on the same small, residual segment(s) of non-nucleosomal DNA or RNA that might persist even after the extensive digestion by MNase. Importantly, other components of the 40S hnRNP particle were not identified in the PALB2/BRCA nucleoprotein complex, indicating that the binding is specific to hnRNP C and that hnRNP C has functions outside of the 40S particle. Again, no difference in hnRNP C abundance was observed in the complexes purified after DNA damage (Fig. 1D).

To test whether hnRNP C and PALB2 interact with each other, we immunoprecipitated (IPed) endogenous PALB2 or hnRNP C from whole cell lysates, but no co-IP of the other protein was detected (not shown). We also overexpressed and IPed GFP- or FLAG-HA-double tagged versions of hnRNP C from whole cell lysates but also failed to detect any co-IP of PALB2 (not shown). Thus, it is unlikely that hnRNP C and PALB2 interact in cell lysates in a significant manner. To confirm the association of endogenous PALB2 and hnRNP C in the chromatin fraction and further test if the association is DNA- or RNA-mediated, we digested the insoluble nuclear materials from U2OS cells with either DNase I or RNase A and then IPed PALB2 from the solubilized fractions. As shown in Fig. 1E, co-IP of hnRNP C and PALB2 was detected in RNase A-released fraction even though this fraction contained less PALB2, suggesting that the association between the two proteins may be mediated by RNA.

### Depletion of hnRNP C reduces HR and alters DSBR pathway choice

**Figure 2:**
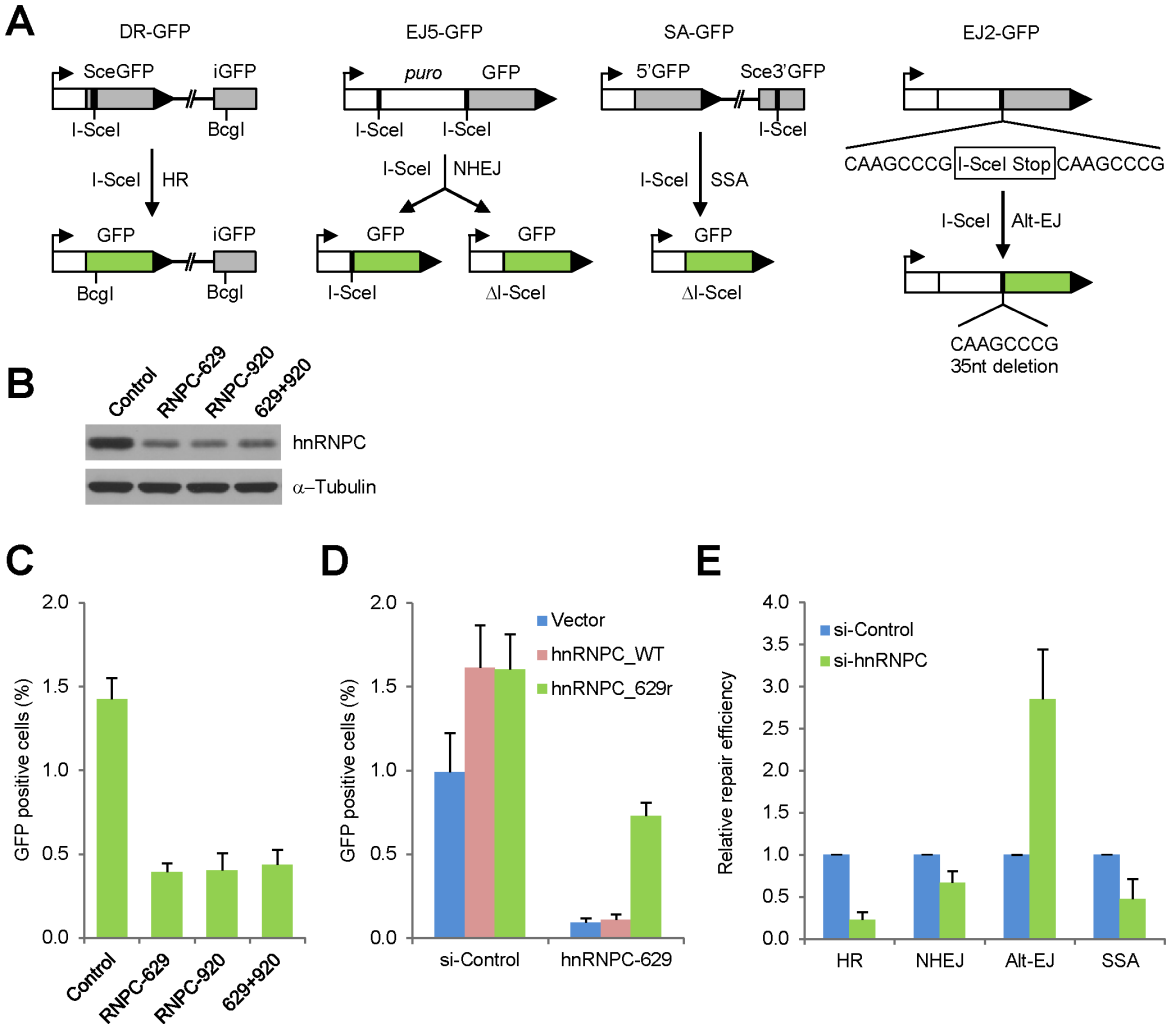
Critical role of hnRNP C in HR and DSBR. **A.** Schematic diagrams of the GFP-based DNA repair reporters used in this study. **B**–**C**. DR-U2OS cells containing a stably integrated HR reporter were treated with control or hnRNP C siRNAs for 48 hr and then transfected with an I-SceI expression plasmid (pCBASce) to induce DSB formation and repair. **B** shows representative downregulation of hnRNP C 72 hr after siRNA transfection and **C** shows GFP positive cells measured 60–72 hr after pCBASce transfection. **D**. DR-U2OS cells were treated with control or hnRNP C (629) siRNAs for 72 hr and then co-transfected with pCBASce together with vector, wt hnRNP C or siRNA-resistant hnRNP C plasmids; GFP positive cells were counted 72 hr later. **E**. U2OS cell lines each harboring a different reporter as indicated were treated with control siRNA or a mixture of the two hnRNP C siRNAs for 48 hr and then transfected with pCBASce, and GFP positive cells were measured 72 hr later. Values shown are averages of at least 3 independent experiments and errors bars represent standard deviations.

The existence of hnRNP C in the chromatin-bound PALB2/BRCA complex raises the immediate question whether it functions in HR, a process in which PALB2 and BRCA1/2 play critical roles. To address this question, we used DR-U2OS cells stably integrated with a single copy of a GFP direct repeat HR reporter (Fig. 2A) [13], [30]. Under normal conditions, GFP expression does not occur since the two GFP genes are either mutated or incomplete. Upon expression of I-SceI, the first GFP gene is cleaved yielding a double strand break which may subsequently be repaired by HR, NHEJ or single strand annealing (SSA). HR-mediated repair using the second GFP gene as a template would lead to restoration of a functional GFP open reading frame (ORF) and therefore GFP-positive cells which can be quantified by Fluorescence-activated Cell Sorting (FACS).

We silenced hnRNP C expression in DR-U2OS cells using two different siRNA sequences, both individually and in combination, and found that depletion of hnRNP C down-regulated HR by over 3 fold (Fig. 2B–C) as compared with control siRNA-treated cells. To rule out the possibility that the down-regulation of HR was caused by siRNA off-target effect, we generated an siRNA-resistant hnRNP C cDNA construct, by introducing 4 silent mutations in the target sequence of siRNA 629 (Fig. S1), and tested if it could reverse the knockdown of HR efficiency by the siRNA. As shown in Fig. 2D, the siRNA-immune cDNA largely restored HR rate whereas the wild type cDNA did not show any effect, indicating that the reduction of HR following the siRNA treatment was specifically due to loss of hnRNP C.

Next, we asked if the loss of hnRNP C also impacts other mechanism of DSBR, including non-homologous end joining (NHEJ), single strand annealing (SSA) and alternative end joining (Alt-EJ, also called MMEJ for microhomology-mediated end joining). To this end, we depleted hnRNP C in a series of similar U2OS cell lines each containing a single copy of the respective reporter construct (Fig. 2A) [31], and measured the efficiency of each repair mechanism. As shown in Fig. 2E, loss of hnRNP C altered the efficiency of all 3 other DSBR mechanisms in addition to HR (note that in this experiment a different line of the HR reporter cells were used). While NHEJ was impacted only moderately, a 3-fold increase in the rate of Alt-EJ and a 2-fold reduction of SSA rate were observed. The potential cause of these changes is discussed later.

### Loss of hnRNP C impairs cellular response to ionizing radiation (IR)

**Figure 3:**
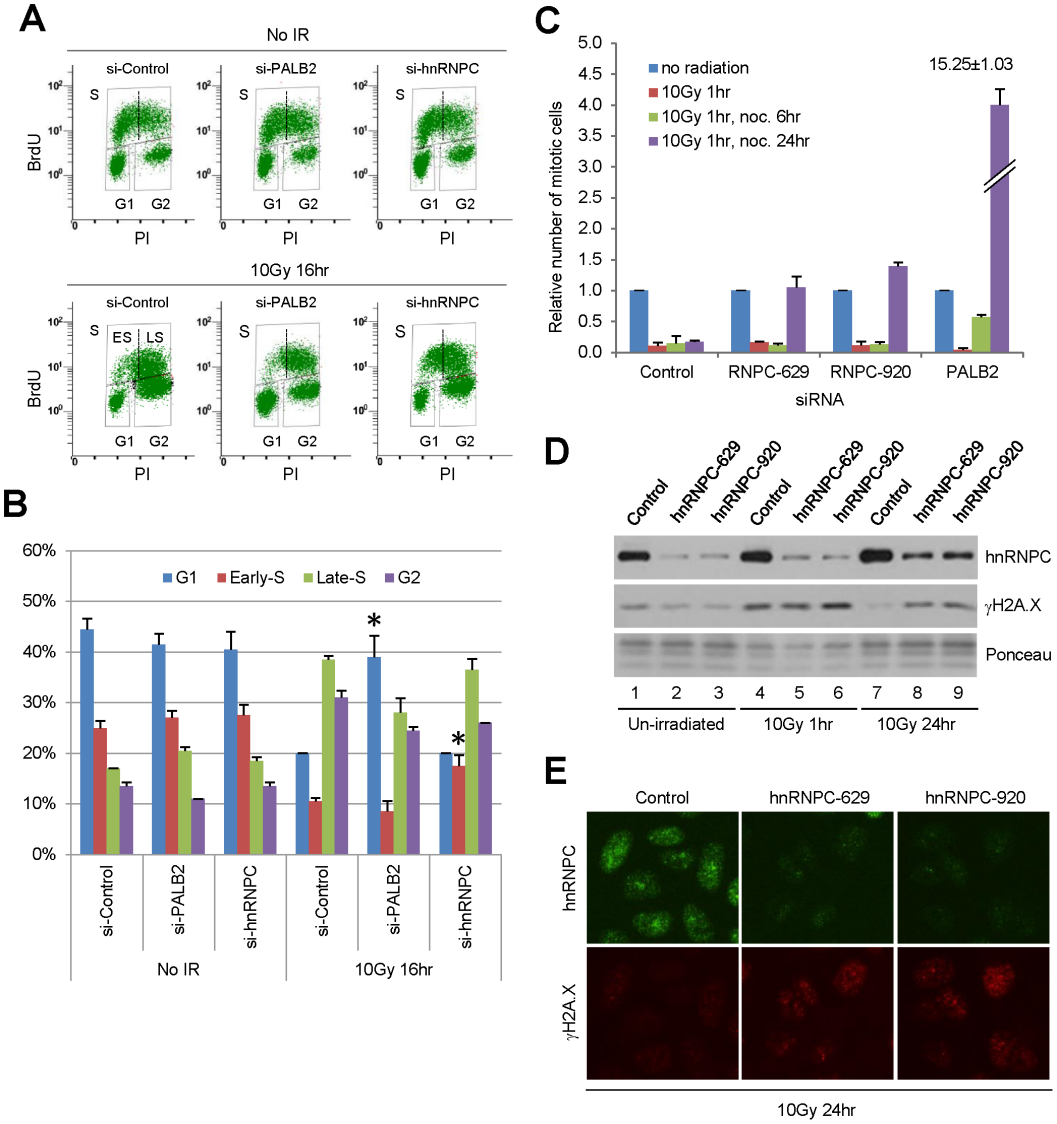
Effect of hnRNP C depletion on cell cycle distribution before and after IR. **A**. DR-U2OS cells were treated with control, PALB2 or hnRNP C siRNAs for 72 hr and then subjected to 10 Gy of IR. Cells were labeled with BrdU either before or 16 hr post IR, and cell cycle profiles were analyzed by anti-BrdU staining and FACS. Cells in S, G1 and G2/M phases were indicated by upper, lower left and lower right boxes, respectively. Early S and late S phase cells are separated by an arbitrary dotted line and indicated by “ES” and “LS”. **B**. Quantification of cell cycle distributions in two independent experiments. Error bars represent standard deviations, and the asterisk indicates p≤0.05. **C**. Cells were treated with the siRNAs and subjected to IR in the same way as in A, and mitotic index was measured by phosphorylated histone H3 staining and FACS. **D.** Cells were treated with control or hnRNP C siRNAs for 72 hr and then subjected to 10 Gy of IR. Cells were harvested at indicated time points, and cellular abundance of hnRNP C and γH2A.X were analyzed by Western blotting. **E.** Cells treated with siRNAs and IR as in **D** were fixed and the abundance and localization of hnRNP C and γH2A.X were analyzed by IF.

Given the important role of hnRNP C in HR and DSBR pathway choice, we analyzed the effect of hnRNP C loss on cell cycle distribution and progression before and after IR by 5-bromo-2′-deoxyuridine (BrdU) incorporation. In addition to a non-targeting control siRNA, a PALB2 siRNA was also used as a reference for DNA damage-induced cell cycle checkpoints, as PALB2 plays important roles in both S phase and G2/M checkpoints [13], [32]. Although cells depleted of hnRNP C consistently grew slower, there was practically no difference in cell cycle distribution before DNA damage suggesting that cells in all cycle phases were almost equally affected (Figs. 3A–B and S2). This finding also implies that the strong HR defect and the changes in other repair mechanisms caused by hnRNP C knockdown were not due to a lack of cells in S and G2 phases which would preclude HR from occurring.

Six hours post IR (10Gy), control cells showed a marked reduction of DNA synthesis that was especially pronounced in late S phase population (Fig. S2), indicative of an active S phase checkpoint. Cells treated with PALB2 siRNA displayed a clearly milder reduction of BrdU incorporation in the late S phase compared to control cells, reflecting a defect in the S phase checkpoint as we reported previously [13]. Interestingly, inhibition of DNA synthesis in all S phase cells was seen in hnRNP C-depleted cells (Fig. S2B). Sixteen hours post IR, the S phase population of control cells had mostly reached late S phase, and PALB2-depleted cells had progressed even further as can be judged by a reduction of S phase population and an increase in the following G1 (Figs. 3A–B and S2C). This behavior of PALB2-depleted cells reflected defects in both S phase and G2/M checkpoints [13], [32]. In contrast, S phase progression in hnRNP C-depleted cells appeared to be slower, as a significant population of cells remained in the early S phase at this point, indicating that hnRNP C plays a role in the recovery of DNA synthesis and S phase progression after DNA damage.

Since PALB2 and BRCA proteins play important roles in the G2/M checkpoint maintenance, we further analyzed the mitotic index of the cells before and after IR. As shown in Fig. 3C, cells depleted of hnRNP C showed an equally dramatic drop of mitotic index as did control cells shortly (1 hr) after IR, which lasted at least until 6 hr. By 24 hr after IR, still no mitotic entry had occurred in control cells, whereas a small number of cells depleted of hnRNP C had escaped the G2/M checkpoint and had been collected in mitosis by nocodazole. In contrast, depletion of PALB2 elicited a profound defect in checkpoint maintenance as reflected by a clear checkpoint escape that already started at 6 hr and a highly significant collection of mitotic cells at 24 hr post radiation. Note that without nocodazole cells would have proceeded through mitosis and accumulated in G1, as shown in Fig. 3A–B. These results demonstrate that loss of hnRNP C does not affect the activation of the G2/M checkpoint but elicits a small defect in checkpoint maintenance. Combined with the fact that an equal number of or more hnRNP C-depleted cells were in the G1 phase than control cells post IR (Figs 3B and S2C), the results further suggest that loss of the protein does not impair the G1/S checkpoint.

To test the importance of hnRNP C in the overall DSB repair efficiency after IR, hnRNP C-depleted cells were irradiated and analyzed for the induction and persistence of phosphorylated histone H2A.X (γH2A.X), a marker of DSBs, post damage. At 1 hr post IR, hnRNP C-depleted cells showed similar induction of γH2A.X compared with control cells (Fig. 3D), indicating that the initial induction of DSBs by IR is hnRNP C-independent. However, 24 hr post IR, while γH2A.X abundance had returned to the pre-damage level in control cells, significant persistence of γH2A.X in hnRNP C-depleted cells was observed by both Western blotting and immunofluorescence (IF) (Fig. 3D–E). These results appear to indicate that hnRNP C loss causes a significant deficit in overall repair efficiency of DSBs. To confirm this possibility, we further carried out the comet assay, a more direct analysis of DNA repair. Surprisingly, only small increases in the number and mean length of comet tails were observed (at 22 hr post IR) in cells depleted of hnRNP C (Fig. S3). This result indicates that the overall DSB repair efficiency is only slightly reduced in the absence of the protein and that the increased Alt-EJ may have compensated for the substantial reduction of HR and SSA. Since the small differences in overall repair efficiency revealed by the comet assay may not fully explain the more evident contrast in γH2A.X abundance between the cells at 24 hr post IR, hnRNPC may also regulate, likely indirectly, the dephosphorylation of γH2A.X during the recovery phase after DNA damage.

### Localization of hnRNP C to DNA damage sites

**Figure 4:**
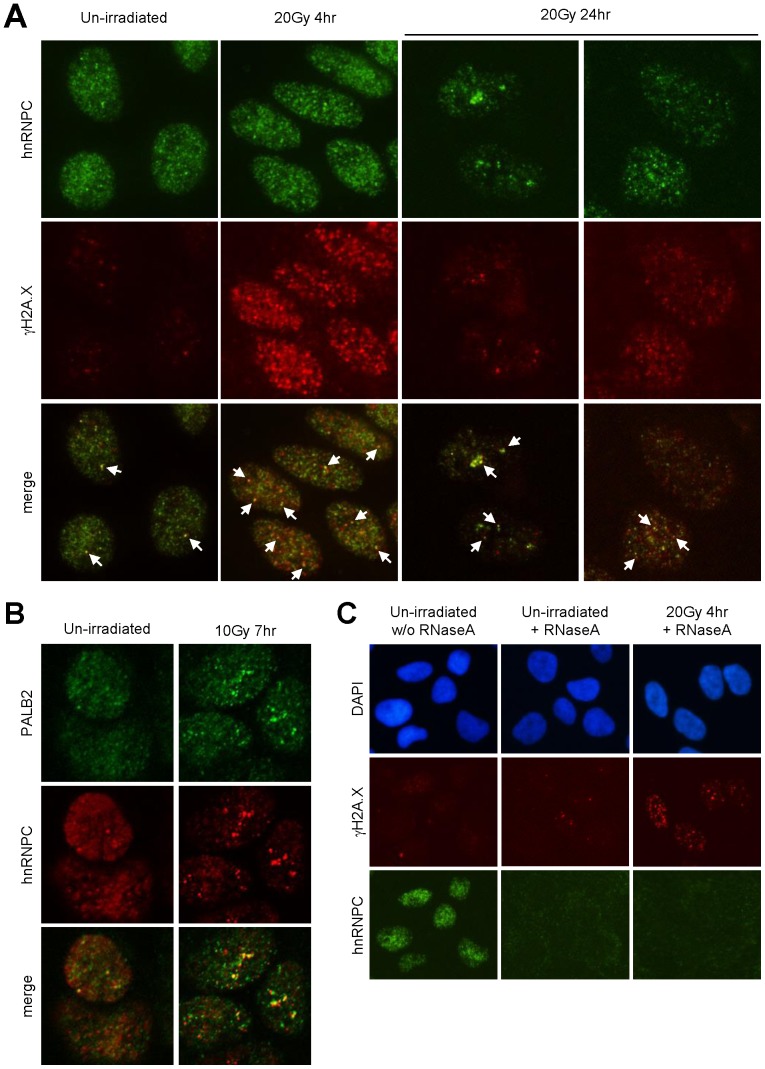
Nuclear localization properties of hnRNP C. **A.** Control and irradiated DR-U2OS cells as indicated were fixed, permeabilized and double stained with hnRNP C and γH2A.X antibodies. Some of the nuclear foci where the two proteins colocalize are marked by white arrows. **B.** Control and irradiated cells were fixed, permeabilized, co-stained with hnRNP C and PALB2 antibodies, and analyzed by confocal microscopy. **C.** Control and irradiated cells were first permeabilized, then treated without or with RNase A and finally fixed for IF analysis.

We further asked if the protein is recruited to DSBs following DNA damage by IF. In un-irradiated cells, hnRNP C showed a largely pan-nuclear staining pattern with varying numbers of distinct foci (Fig. 4A). Interestingly, colocalization of hnRNP C and γH2A.X, mostly at just one round focus per cell, was observed. At 4 hr after IR, there was a slight but discernible change in the staining pattern of hnRNP C, with somewhat more foci having been formed and their shapes becoming more irregular. At the same time, hnRNP C was found to colocalize with γH2A.X, which had been dramatically induced, in at least several distinct foci per cell. At 24 hr post IR, a substantial decrease of overall hnRNP C staining signals was noticed (Fig. 4A). Interestingly, two populations of cells were observed with respect to hnRNP C staining pattern, one with bright and often clustered hnRNP C foci and the other with weaker and dispersed foci. At this time, partial colocalization of hnRNP C with the residual γH2A.X was still present, especially in cells with bright and clustered hnRNP C foci. Next, we tested if hnRNP C co-localizes with PALB2. Confocal microscopy images revealed a partial co-localization between the proteins which was present already in undamaged cells and was further increased after IR (Fig. 4B). These data demonstrate a dynamic relocalization of hnRNP C after DNA damage and a limited but clear presence of the protein at sites of damage.

Being an RNA-binding protein, hnRNP C may also be able to bind ssDNA, which plays essential roles in DNA damage signaling and the initiation of HR [33], [34]. Therefore, we asked if hnRNP C may bind ssDNA at DNA damage sites, such as resected DSB ends. To this end, unfixed cells were first permeabilized, cellular RNAs were then removed by RNase A digestion, and the localization of hnRNP C was examined by IF. Remarkably, RNase A digestion completely eliminated the hnRNP C staining signal in both control and irradiated cells (Fig. 4C), suggesting that the binding of hnRNP C to chromatin and/or nuclear structures may be entirely RNA-dependent. The fact that γH2A.X foci still remained post RNase A treatment indicate that the digestion was specific to RNA and that all visible hnRNP C foci were formed in an RNA-dependent manner.

### hnRNP C selectively regulates the expression of key HR and repair genes

**Figure 5:**
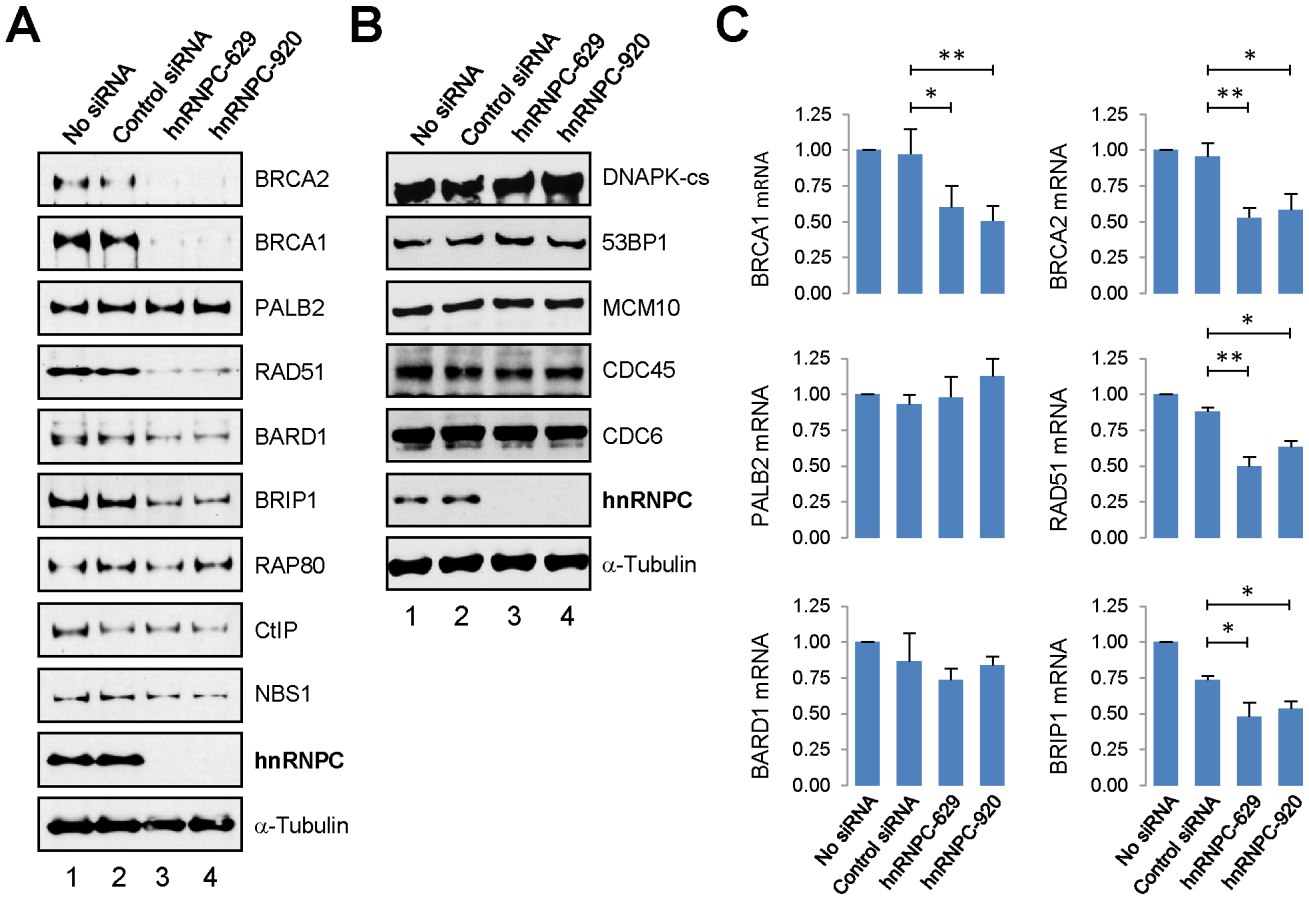
Selective regulation of DNA repair and replication genes by hnRNP C. **A**–**B.** DR-U2OS cells were treated with transfection reagent alone (labeled as “no siRNA”), control siRNA or hnRNP C siRNAs for 72 hr and protein amounts were analyzed by Western blotting. **C.** Total RNAs were isolated from cells 48–72 hr after transfection and mRNA amounts of the 6 genes indicated were analyzed by quantitative RT-PCR. Values shown are averages of at least 3 independent experiments and error bars represent standard deviations. P values were calculated with student's t test using GraphPad Prism V5. P values smaller than 0.05 are denoted by one asterisk and those smaller than 0.01 are indicated by two asterisks.

The limited and RNA-dependent localization of hnRNP C to DNA damage sites makes it unlikely that the protein directly participates in HR by binding to ssDNA intermediates generated during the process. Thus, we asked if hnRNP C regulates the expression of key HR genes. Interestingly, we observed in hnRNP C knockdown cells strongly reduced protein levels of BRCA1, BRCA2, RAD51 and BRIP1 (Fig. 5A). For BRCA1 and RAD51, the much reduced abundance and loss of foci formation were also confirmed by IF (Fig. S4). Additionally, levels of BARD1 and perhaps NBS1 were also lower. In contrast, cellular abundance of PALB2, RAP80, CtIP and NBS1 was not affected. The apparently selective effect of hnRNP C on the expression of the above HR-related genes prompted us to further examine a panel of key NHEJ and DNA replication factors following its depletion. As shown in Fig. 5B, levels of NHEJ proteins DNAPK and 53BP1 were unchanged, as were the amounts of essential DNA replication factors MCM10, CDC45 and CDC6, consistent with the largely unaffected cell cycle distribution under the condition used. These findings suggest that the remarkable HR defect of hnRNP C-depleted cells is, at least in part, due to greatly reduced concentrations of above-noted key HR regulators.

Next, we measured mRNA amounts of *BRCA1*, *BRCA2*, *PALB2*, *RAD51*, *BARD1* and *BRIP1* in control and hnRNP C-depleted cells. As shown in Fig. 5C, hnRNP C depletion resulted in significant reduction of *BRCA1*, *BRCA2*, *RAD51* and *BRIP1* mRNAs, while the *PALB2* messenger was slightly upregulated. We noticed that transfection of the control siRNA caused modest but consistent decreases in *RAD51*, *BARD1* and particularly *BRIP1* mRNA levels, indicating that a sequence-independent effect of siRNA transfection may be responsible for a fraction of the reduction observed for these genes. Nevertheless, the effect of the hnRNP C-specific siRNAs was significantly stronger.

**Figure 6:**
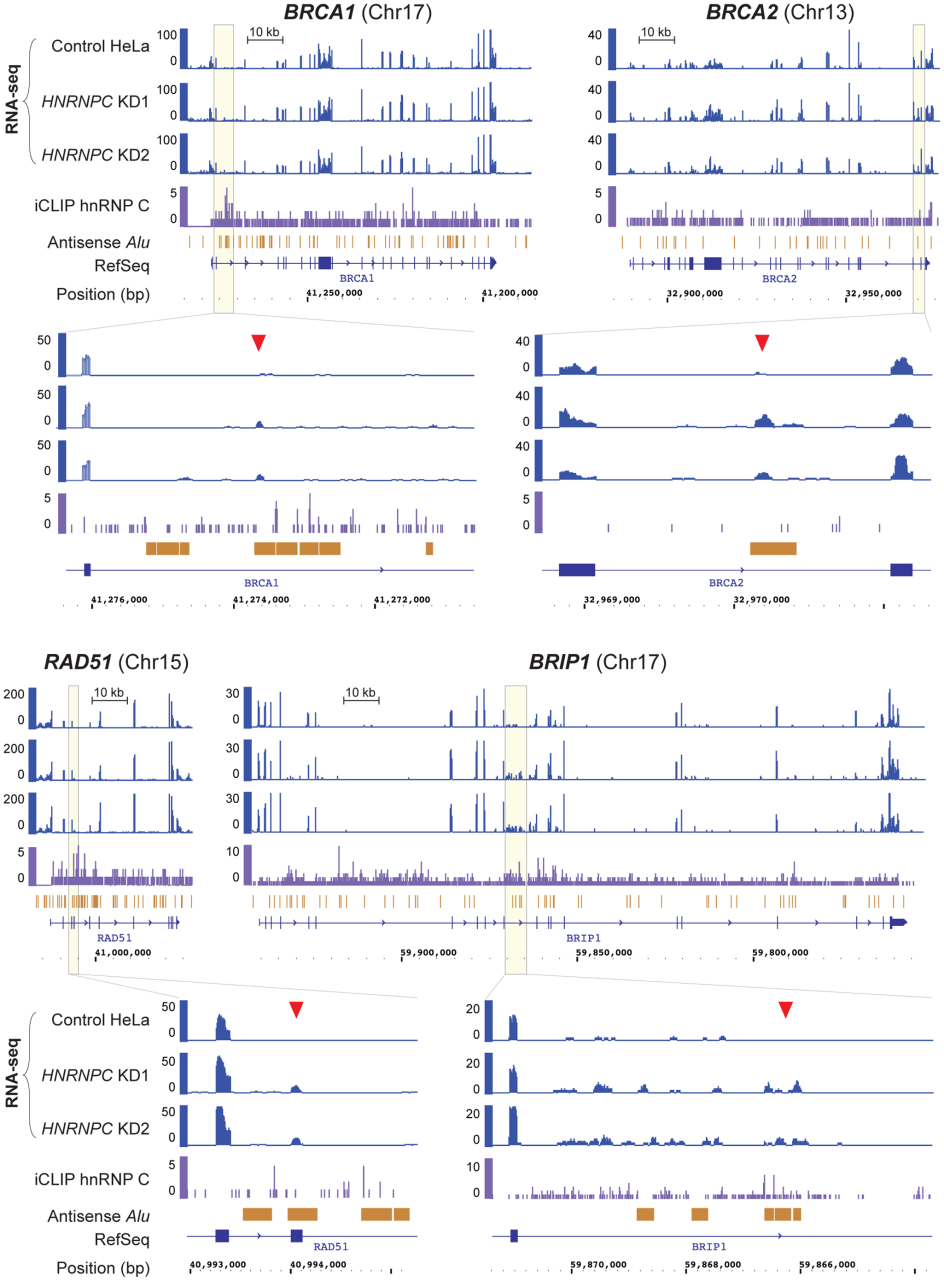
Depletion of hnRNP C leads to *Alu* element exonization in *BRCA1*, *BRAC2*, *RAD51* and *BRIP1*. Genome browser view of *BRCA1*, *BRAC2*, *RAD51* and *BRIP1* genes displaying RNA-Seq data (overlapping reads per nucleotide; blue) from control and *HNRNPC* knockdown HeLa cells, that were independently transfected with two different siRNAs (KD1 and KD2), as well as hnRNP C iCLIP data (crosslink events per nucleotide; purple). RefSeq transcript annotations (blue) and *Alu* elements in antisense orientation (orange) are depicted below. Yellow boxes contain zoomed regions within the four genes where hnRNP C-repressed *Alu* exonization events were detected (marked by red arrowheads). See ref. #8 for details for data generation and analyses.

Finally, we asked if hnRNP C may directly bind the transcripts of the above HR genes and regulate their splicing. To this end, we analyzed the newly generated high resolution iCLIP and RNA-Seq data [8]. As shown in Figs. 6 and S5, iCLIP revealed hnRNP C binding sites in all six genes. Moreover, consistent with the previously described sequence specificity of hnRNP C, binding sites preferentially located on uridine tracts (Fig. S5), indicating that the binding was specific. Interestingly, exonization of *Alu* elements was found in *BRCA1*, *BRCA2*, *RAD51* and *BRIP1* mRNAs following hnRNP C depletion (Fig. 6) but not in those of *PALB2* and *BARD1*. Thus, a correlation exists between the downregulation of mRNA levels and exonization of *Alu* elements after hnRNP C loss. Since exonized *Alu* sequences either contain nonsense codons or result in frameshifts, the aberrantly spliced mRNAs can be expected to be both unproductive and unstable due to nonsense-mediated decay (NMD). Taken together, our results demonstrate that hnRNP C directly binds to transcripts of above key HR genes and regulates their splicing and functionality.

## Discussion

In this study, we found a significant presence of hnRNP C in PALB2-containing nucleoprotein complexes. The association between hnRNP C and PALB2 appeared to be indirect and instead mediated by RNA (Fig. 1E). hnRNP C was found to undergo dynamic changes in intra-nuclear localization after DNA damage and to be recruited to a subset of DNA damage sites where it co-localized with PALB2. RNase A treatment of permeabilized cells completely eliminated nuclear staining signal of hnRNP C, indicating that the protein may bind exclusively to the RNA components of nuclear structures. Depletion of hnRNP C severely compromised HR while increasing the rate of Alt-EJ/MMEJ. In addition, loss of hnRNP C impaired S phase progression after IR. Interestingly, depletion of hnRNP C resulted in a profound decrease of cellular BRCA1/2 and RAD51 protein abundance, which can be attributed, at least in part, to reduced amounts of their mRNAs. These results establish hnRNP C as a DNA damage response factor and an important regulator of HR as well as general DSBR pathway choice.

The pathway choice in DSBR is a competitive process in which the commitment to one mechanism at a given break precludes others. In general, SSA should increase following BRCA2 and/or PALB2 loss as reported before [25], [35], since the resulting inability to commence HR following initial end resection would lead to excessive resection exposing more homologous stretches in single stranded DNA overhangs suitable for annealing. However, BRCA1 loss has been reported to impair SSA [35]. This may be explained by the recent finding that BRCA1, possibly in cooperation with CtIP, promotes resection [23], [36], which is a prerequisite not only for HR but also for SSA. Thus, with respect to SSA, the effect of BRCA1 loss overshadows that of BRCA2 or PALB2 loss. In this vein, the low BRCA1 abundance after hnRNP C loss may in part explain the reduced SSA observed in this study. The likely inability of hnRNP C-depleted cells to fully resect DSB ends for HR and SSA may also help explain the increased use of Alt-EJ. Interestingly, Alt-EJ appears to effectively compensate the impairment of the other three mechanisms since the overall DSBR efficiency is only slightly reduced as revealed by the comet assay (Fig. S3).

The mechanism(s) by which hnRNP C regulates the expression of the *BRCA* and related genes may be complex. As a chaperone of the transcriptome, hnRNP C presumably influences the expression of a large number of genes and therefore may indirectly affect the abundance of *BRCA* gene products through other factors involved in transcription, RNA splicing and stability, or protein synthesis, posttranslational modification and degradation, etc. However, analysis of iCLIP and RNA-Seq data revealed direct binding of hnRNP C to transcripts of all of the 6 genes tested and that loss hnRNP C resulted in exonization of intronic *Alu* sequences in the four genes whose mRNA amounts were affected (Figs. 6 and S5), indicating that hnRNP C also directly regulates the splicing of the transcripts to ensure proper expression of the genes. Considering the large decrease in BRCA1, BRCA2, RAD51 and BRIP1 protein amounts and the relatively moderate reduction of their mRNA levels, it is reasonable to assume that the stability and/or translation of the proteins may have also been negatively affected by hnRNP C loss.

Similar to our findings, a recent whole genome screen for HR regulatory genes by Adamson et al. identified RBMX (hnRNP G) as a novel factor that positively regulates HR and resistance to DNA damage [37]. In the above study, the authors demonstrate that RBMX is critical for cellular BRCA2 protein abundance. In light of our findings, RBMX may regulate BRCA2 expression at the mRNA level. The same screen also found hnRNP C and hnRNP K as potential regulators of HR, whose depletion reduced HR by ∼4 fold and ∼2.5 fold, respectively (but these genes were not specifically studied therein). Such an effect of hnRNP C depletion is similar to what we observed (Fig. 2), and the potential role of hnRNP K in HR is consistent with a reported role of the protein to bind the *BRCA1* promoter [38]. Depletion of other hnRNP proteins produced no or little effect on HR. Thus, given the lack of other hnRNPs in our PALB2 complex, hnRNP C clearly has an important and unique role in regulating the expression of a set of key DNA repair genes that is independent from its general function in the hnRNP particles. Since both proteins are recruited to DNA damage sites, these two studies together suggest the involvement of a RNA regulatory program in regulating the DNA damage response and repair.

Since knockouts of *Brca1*, *Brca2* and *Rad51* in mice all result in embryonic lethality [39]–[42], the much reduced levels of these proteins in the absence of hnRNP C may contribute to the embryonic lethal phenotype of the hnRNP C knockout mice [9]. Also, it has been found that downregulation of the protein in HeLa cells leads to cellular sensitivity to topoisomerase inhibitors camptothecin and ICRF-193 as well as another DNA damaging agent hydrogen peroxide (H_2_O_2_) [43]. In particular, camptothecin generates single strand breaks (SSBs) in the S phase that eventually lead to replication fork collapse (DSB formation) which requires HR for repair [44]. Our finding that hnRNP C is critical for HR may, at least in part, explain the sensitivity of hnRNP C-depleted cells to the above DNA damaging agents.

hnRNP C undergoes a dynamic relocalization after ionizing radiation and localizes to a subset of DNA damage sites (Fig. 4A), indicating that the protein may actively participate in the DNA damage response. The fact that all of its nuclear staining signals were eliminated by RNase A treatment (Fig. 4C) implies an exclusive RNA-dependent association of the protein with nuclear structures, and the extensive nuclear staining signal of hnRNP C in pre-permeabilized cells also indicates that much of the protein is bound to nascent RNAs that are still attached to the chromatin. These observations together suggest that hnRNP C may respond to DNA (or RNA) damage by altering the normal schedule of nascent RNA processing (or transcription) to ensure faithful expression of genetic information. Finally, the association between hnRNP C and PALB2-BRCA1/2 proteins appears to be RNA-mediated, and it remains to be seen exactly how the proteins and bound nucleic acids work together to promote proper repair of DNA damage or faithful gene expression after radiation.

## Materials and Methods

### Cell lines, siRNA and transfections

DR-U2OS as described before [13] were used in all experiments except PALB2 tandem affinity purification. Cells were cultured in Dulbecco's Modified Eagle's Medium (DMEM) supplemented with 10% FBS in a humidified chamber containing 5% CO_2_ at 37°C. HeLa S3 cells were used for PALB2 purification and were grown in Minimum Essential Medium Eagle (MEM) (Sigma, M8028) supplemented with 5% FBS in spinner flasks in a 37°C warm room.

For general siRNA knockdown experiments, cells were plated at ∼60% confluence in 6 cm dishes and siRNAs were transfected using Lipofectamine RNAiMax (Invitrogen) at 5 nM final concentration. Unless otherwise mentioned, cells were trypsinized and reseeded into 6-well (for Western and FACS) or 12-well plates (for IF) 48 hr after transfection and various analyses were carried out another 24 hr later. The sense strand sequences of siRNAs used are: control, UUCGAACGUGUCACGUCAAdTdT; hnRNP C-629, CAACGGGACUAUUAUGAUAdTdT; and hnRNP C-920, GUAGAGAUGAAGAAUGAUAdTdT.

### Purification of PALB2 complexes

HeLa S3 cells harboring the empty vector or stably expressing tagged PALB2 were described before [18]. The cells were harvested, washed with PBS and permeabilized with 10 volumes of MNase buffer I (20 mM Tris-HCl [pH 7.5], 100 mM KCl, 0.3 M sucrose, 0.1% Triton X-100, 10 mM NaF, 1 mM sodium orthovanadate, with Complete® protease inhibitor tablet (Roche)) by rocking at 4°C for 20 min. Nuclear structures were harvested by centrifugation at 5,000 rpm for 10 min, washed with 10 volumes of MNase buffer I, and then resuspended in 2 volumes of MNase buffer II (20 mM Tris-HCl [pH 7.5], 100 mM KCl, 2 mM CaCl_2_, 0.3 M sucrose, 0.1% Triton X100, 10 mM NaF, 1 mM sodium orthovanadate, with Complete® EDTA-free protease inhibitor tablet) containing MNase at a final concentration of 3 u/µl. The nuclei were digested by rocking at room temperature for 90 min. The reactions were stopped by adding EGTA and EDTA to 5 mM each, and supernatants containing solubilized chromatin were collected by centrifugation at 5,000 rpm for 10 min. Nucleoprotein complexes containing the FLAG-HA double tagged PALB2 were isolated by tandem affinity purification as previously described [18].

For analysis of DNA fragment size after MNase digestion, solubilized fractions were treated first with 5 µg/100 µl RNase A at 37°C for 30 min and then with 100 µg/ml proteinase K in the presence of 0.5% SDS at 55°C overnight. Digested samples were extracted with phenol/chloroform, and then analyzed on a 1.5% agarose gel.

### Antibodies, Western blotting and immunoprecipitation

The anti-PALB2 M10 and M11 antibodies used in this study were raised in rabbits against GST-fusions of 1–120aa and 601–880aa of human PALB2, respectively, and affinity purified. The rabbit BRIP1 antiserum is a gift from Dr. Sharon Cantor (University of Massachusetts). Other antibodies used are as follows: hnRNP C (Santa Cruz, sc-15386 and sc-32308), γH2AX (Millipore, #05-636), BRCA1 (Millipore, #07-434), BRCA2 (EMD Biosciences, OP95), RAD51 (Santa Cruz, sc-8349), BARD1 (Santa Cruz, sc-11438), RAP80 (Bethyl Labs, A300-763A), CtIP (Bethyl Labs, A300-488A), NBS1 (Bethyl Labs, A300-290A), DNAPK-cs (Bethyl Labs, A300-516A), 53BP1 (Bethyl Labs, A300-272A), MCM10 (ProteinTech Group, #12251-1-AP), CDC6 (Epitomics, #3561-1), CDC45 (Epitomics, 3840-1), α-Tubulin (Sigma, T9026).

For Western analysis, cells were lysed in NETNG400 (20 mM Tris-HCl [pH 7.4], 400 mM NaCl, 0.5 mM EDTA, 0.5% NP-40 and 10% glycerol) buffer for 10 min with mixing at 4°C. Lysates were clarified by centrifugation at 21,000×*g* for 10 min at 4°C. Supernatants were collected and protein concentration was measured using Bradford's assay (Bio-Rad). Equal amounts (15–20 µg) of proteins were subjected to SDS-PAGE. Proteins were then immobilized onto nitrocellulose membranes followed by incubations in primary and secondary antibodies. Detection was carried out using Enhanced Chemiluminescence (ECL) detection system (GE Healthcare).

For immunoprecipitation of endogenous PALB2 from nuclease-released nuclear fractions, low salt-resistant nuclear structures were prepared from DR-U2OS cells with MNase buffer I, digested with DNase I or RNase A in MNase buffer II, and the released proteins (supernatants) were subjected to IP with the anti-PALB2 M11 antibody. Approximately ∼4×10^6^ cells were used for each IP. The digestion was for 45 min at room temperature with 50 units of DNase I or 30 µg of RNase A in a volume of 300 µl, and the IP was carried out for overnight at 4°C.

### Homologous recombination assays

In experiments shown in Fig. 2B–C, DR-U2OS HR reporter cells were seeded at 150,000 cells per well into 6-well plates the day before transfection. On day one, cells were transfected with siRNAs using Oligofectamine (Invitrogen) as per the manufacturer's instruction. On day two, medium was replaced with fresh medium containing serum and antibiotics. On the third day, cells were transfected with pCBAsce plasmid (2 µg/well) using FuGENE® 6 transfection reagent (Roche). Cells were trypsinized on the sixth day, resuspended in 0.5 ml PBS and GFP-positive cells were scored using a Beckman Coulter FC-500 flow cytometer. In the HR reconstitution assay shown in Fig. 2D, DR-U2OS cells plated in 10 cm dish (1.2×10^6^ cells per dish) were transfected with siRNAs using Lipofectamine RNAiMax. 24 hr later, cells were split into 6-well plates (200,000 cells per well). On the next day, cells were co-transfected with the same siRNA, pCBAsce (750 ng) and hnRNP C expression vectors (750 ng) using Lipofectamine 2000. Medium was refreshed 8 hr later and GFP positive cells were counted 72 hr post co-transfection. DNA repair assays shown in Fig. 2E were carried out as in 2B–C except that RNAiMax was used for siRNA transfections. The final concentration of siRNAs was 8 nM in all above experiments.

### Immunofluorescence

Cells were seeded into 12-well plates containing 15 mm #1 round coverslips the day before treatment or analyses. Briefly, cells were fixed with 3% (w/v) paraformaldehyde (in PBS with 300 mM sucrose) for 10 min at room temperature, permeabilized with 0.5% Triton X-100 (in PBS) and then sequentially incubated with primary and secondary antibodies (diluted in PBS containing 5% goat serum) for 1 hr each at 37°C. Each of the above steps was followed by three PBS washes. After staining, coverslips were mounted onto glass slides with VECTASHIELD mounting medium with DAPI (Vector Labs) and observed using a Nikon Eclipse Ti fluorescent microscope.

For analyzing the RNA dependence of hnRNP C nuclear localization, cells were first permeabilized with CSK buffer (20 mM HEPES [pH 7.4], 300 mM sucrose, 3 mM MgCl_2_, 50 mM NaCl, 0.5% Triton X-100) for 4 min and then treated with 100 µg/ml of RNase A (Sigma) for 10 min at room temperature. Cells were then fixed with 3% paraformaldehyde and stained as described above.

### Cell cycle analysis

For cell cycle and DNA synthesis analyses, DR-U2OS cells were treated with siRNAs for 72 hr and then pulse-labeled (in 6-well plates) with 10 µM BrdU for 10 min prior to harvest by trypsinization. Collected cells were washed with PBS and fixed with 10 volumes of ice-cold 70% ethanol and placed at −20°C overnight. Cells were then stained using FITC-conjugated anti-BrdU (BD Biosciences, #347583) following manufacturer's instructions. After staining, cells were pelleted and resuspended in 0.5 ml of PBS solution containing 0.02% (w/v) propidium iodide (PI, Sigma) and 200 µg/ml RNase A, and incubated for 15 min at 37°C. Finally, cells were analyzed by FACS on a Beckman Coulter FC-500 flow cytometer and using the CXP software. To measure mitotic index, cells plated in 10 cm dish were transfected with siRNAs for 48 hr and split into 6-well plates at 750,000 cells per well. On the next day, cells were irradiated and collected at indicated time points. When nocodazole was used, it was added 1 hr after radiation to a final concentration of 100 ng/ml. Harvested cells were fixed with 70% ethanol overnight and stained with anti-phosphorylated histone H3 (pSer10) (Cell Signaling Technology, #9701) following standard protocols. Following the staining cells were incubated with PI and then analyzed by FACS.

### Quantitative reverse transcriptase-PCR (qRT-PCR)

Total RNAs were extracted using RNeasy Plus Mini kit (QIAGEN), and cDNAs were generated using the SureScript® III First Strand Synthesis System (Invitrogen). Real-time PCR was performed using Brilliant II SYBR® Green qPCR Master Mix (Agilent Technologies) on a MX3005P system (Stratagene) using the following parameters: 15 min initial heating (denaturation and hot start enzyme activation) at 95°C, 40 cycles of amplification (95°C for 10 sec and 60°C for 30 sec) followed by melting curve measurement. Data presented are relative mRNA levels normalized to that of RPLP0, with the value in the control group (transfection reagent only) set as 1. Experiments were performed in triplicates for at least 3 times.

Primers were synthesized by Sigma. The sequences of the primers are as follows: RPLP0- ATCAACGGGTACAAACGAGTCCT, AGGCAGATGGATCAGCCAAGAAG; BRCA1- GAATGGATGGTACAGCTGTGTG, ATGGAAGCCATTGTCCTCTGTC; BRCA2- GCCACTTTCAAGAGACATTCAACA, GTACAGTCTTTAGTTGGGGTGGA; PALB2- TGTGATGCTGTACTGTCTTCCTC, GCAATTGTTCCAGAAGTCAAGAT; RAD51- TGTTTGGAGAATTCCGAACTG, GTCAATGTACATGGCCTTTCCT; BARD1- ATTGCTGCTACCAGAGAAGAATG, ACAGCCCACTGCCTATAAGTACA; and BACH1- CAGAAAGGAGAAAAATGATCCAG, CTTTGTTTGTTTGTTGAAAGTTGG.

## Supporting Information

Figure S1 **Construction and expression of an siRNA-resistant form of hnRNP C cDNA expression vector.** A. Silent mutations introduced into the target sequence of the hnRNP C siRNA (RNPC-629). Shown on top is the sequence of the sense primer used for mutagenesis containing 4 silent mutations that would render the cDNA resistant to the siRNA. The bottom sequence is of the wt cDNA with the siRNA target sequence shown in red. The corresponding protein sequence is also shown. B. The modified hnRNP C expression vector was co-transfected, in parallel with the empty vector and the wt expression vector, with pCBASce into DR-U2OS cells. Cells were fixed 48 hr after transfection and IF was conducted using the indicated antibodies.
(PDF)

Figure S2 **Effect of hnRNP C depletion on cell cycle distribution before and after IR.** DR-U2OS cells were treated with control, PALB2 or hnRNP C siRNAs for 72 hr and then subjected to 10 Gy of IR. Cells were labeled with BrdU either before (A) or at 6 and 16 hr post IR (B and C, respectively), and cell cycle profiles were analyzed by anti-BrdU staining and FACS. Cells in S, G1 and G2/M phases were indicated by upper, lower left and lower right boxes, respectively. Numbers in the boxes indicate the percentages of cells in the corresponding phases. In the left panel of B, early S and late S phase cells are indicated by "ES" and "LS" and separated by an arbitrary dotted line.
(PDF)

Figure S3 **Comet assay of hnRNP C-depleted cells after IR.** A. DR-U2OS cells were treated with control or hnRNP C (1∶1 mix of 629 and 920) siRNAs for 72 hr and then subjected to 10 Gy of IR. Cells were harvested at indicated time points following IR and subjected to alkaline comet assay (Trevigen) following manufacturer's instructions. B. Number (in percentage) of cells with comet tails in a representative experiment. C. Mean length of comet tails in a representative experiment. D–G. Length distribution of comet tails in a representative experiment. Comet measurements were carried out using the Image J software, and approximately 100 comets were measured for each condition.
(PDF)

Figure S4 **Reduced abundance and impaired focus formation of BRCA1 and RAD51 in hnRNP C-depleted cells.** Control treated or hnRNP C-depleted DR-U2OS cells were subjected to 10 Gy of IR. Cells were fixed at indicated time points and stained for BRCA1 (A) or RAD51 (B) together with γH2A.X. The antibody used were anti-BRCA1 (#07-434, Millipore), anti-RAD51 (sc-8349, Santa Cruz) and anti-γH2A.X (#05-636, Millipore).
(PDF)

Figure S5 **Binding of hnRNP C to transcripts of HR genes.** A. Genome browser view of PALB2 and BARD1 genes displaying RNA-Seq data (overlapping reads per nucleotide; blue) from control and hnRNP C knockdown HeLa cells, that were independently transfected with two different siRNAs (KD1 and KD2), as well as hnRNP C iCLIP data (crosslink events per nucleotide; purple). RefSeq transcript annotations (blue) and Alu elements in antisense orientation to the shown strand (orange) are depicted below. No Alu exonization events were found in these two genes. B. "Weblogo" showing the base composition at the hnRNP C crosslink sites (position 0) within BRCA1, BRCA2, PALB2, RAD51, BARD1 and BRIP1 gene transcripts as well as the surrounding sequence. The y-axis indicates the informational content for each position in bits. The graph shows the aggregate of all the crosslink sites in the 6 genes.
(PDF)
