# Supplementary material for: Requirement of Heterogeneous Nuclear Ribonucleoprotein C for BRCA Gene Expression and Homologous Recombination
Source: PLoS One. 2013 Apr 9;8(4):e61368. doi: 10.1371/journal.pone.0061368 (PMC3621867; doi:10.1371/journal.pone.0061368)
Supplement: Figure S1 — Construction and expression of an siRNA-resistant form of hnRNP C cDNA expression vector. A. Silent mutations introduced into the target sequence of the hnRNP C siRNA (RNPC-629). Shown on top is the sequence of the sense primer used for mutagenesis containing 4 silent mutations that would render the cDNA resistant to the siRNA. The bottom sequence is of the wt cDNA with the siRNA target sequence shown in red. The corresponding protein sequence is also shown. B. The modified hnRNP C expression vector was co-transfected, in parallel with the empty vector and the wt expression vector, with pCBASce into DR-U2OS cells. Cells were fixed 48 hr after transfection and IF was conducted using the indicated antibodies. (PDF) [file pone.0061368.s001.pdf]

Figure S1 Anantha et al.

A

```
   D  Y  D  F  Q  R  D  Y  Y  D  R  M  Y  S  Y
GGACTATGACTTTCAACGCGATTACTACGATAGGATGTACAGTTACC siR
|||||||||||||||||| || || || |||||||||||||||||||
GGACTATGACTTTCAACGGGACTATTATGATAGGATGTACAGTTACC WT
   D  Y  D  F  Q  R  D  Y  Y  D  R  M  Y  S  Y
```

B

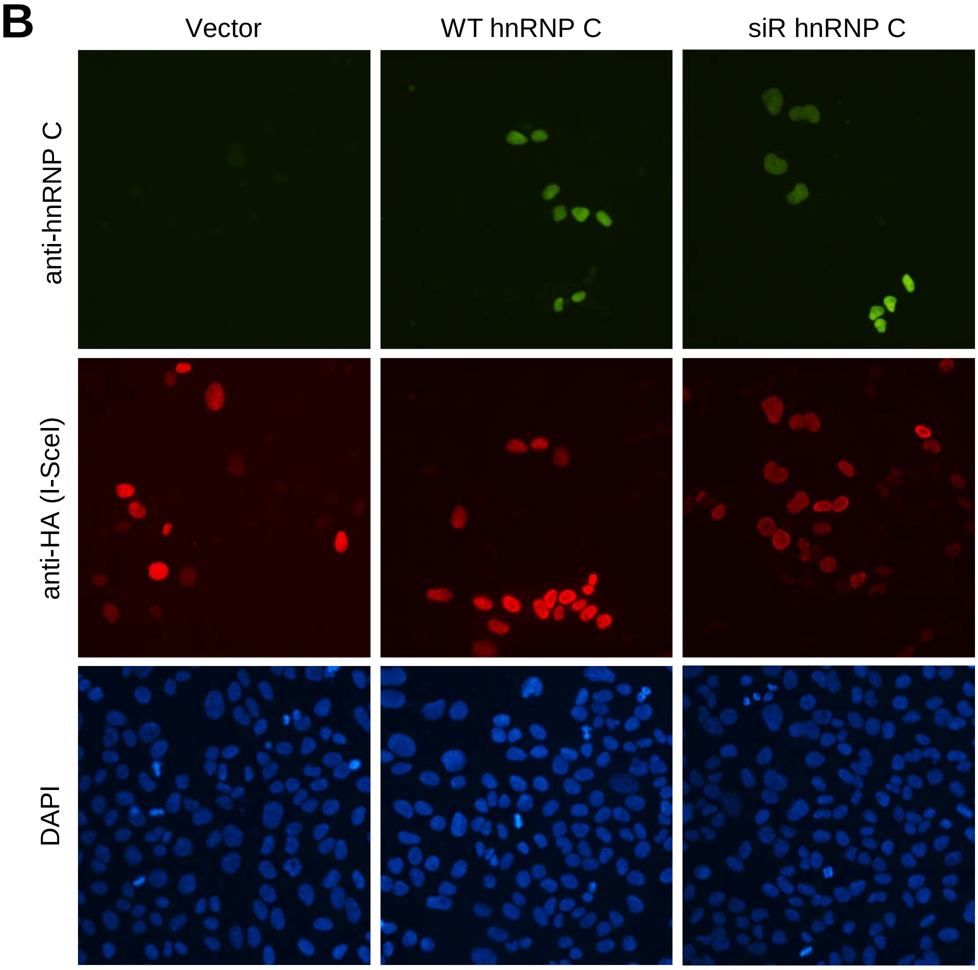

Figure S1. Construction and expression of an siRNA-resistant form of hnRNP C cDNA expression vector. A. Silent mutations introduced into the target sequence of the hnRNP C siRNA (RNPC-629). Shown on top is the sequence of the sense primer used for mutagenesis containing 4 silent mutations that would render the cDNA resistant to the siRNA. The bottom sequence is of the wt cDNA with the siRNA target sequence shown in red. The corresponding protein sequence is also shown. **B**. The modified hnRNP C expression vector was co-transfected, in parallel with the empty vector and the wt expression vector, with pCBASce into DR-U2OS cells. Cells were fixed 48 hr after transfection and IF was conducted using the indicated antibodies.
