# Supplementary material for: Requirement of Heterogeneous Nuclear Ribonucleoprotein C for BRCA Gene Expression and Homologous Recombination
Source: PLoS One. 2013 Apr 9;8(4):e61368. doi: 10.1371/journal.pone.0061368 (PMC3621867; doi:10.1371/journal.pone.0061368)
Supplement: Figure S2 — Effect of hnRNP C depletion on cell cycle distribution before and after IR. DR-U2OS cells were treated with control, PALB2 or hnRNP C siRNAs for 72 hr and then subjected to 10 Gy of IR. Cells were labeled with BrdU either before (A) or at 6 and 16 hr post IR (B and C, respectively), and cell cycle profiles were analyzed by anti-BrdU staining and FACS. Cells in S, G1 and G2/M phases were indicated by upper, lower left and lower right boxes, respectively. Numbers in the boxes indicate the percentages of cells in the corresponding phases. In the left panel of B, early S and late S phase cells are indicated by "ES" and "LS" and separated by an arbitrary dotted line. (PDF) [file pone.0061368.s002.pdf]

Figure S2 Anantha et al.

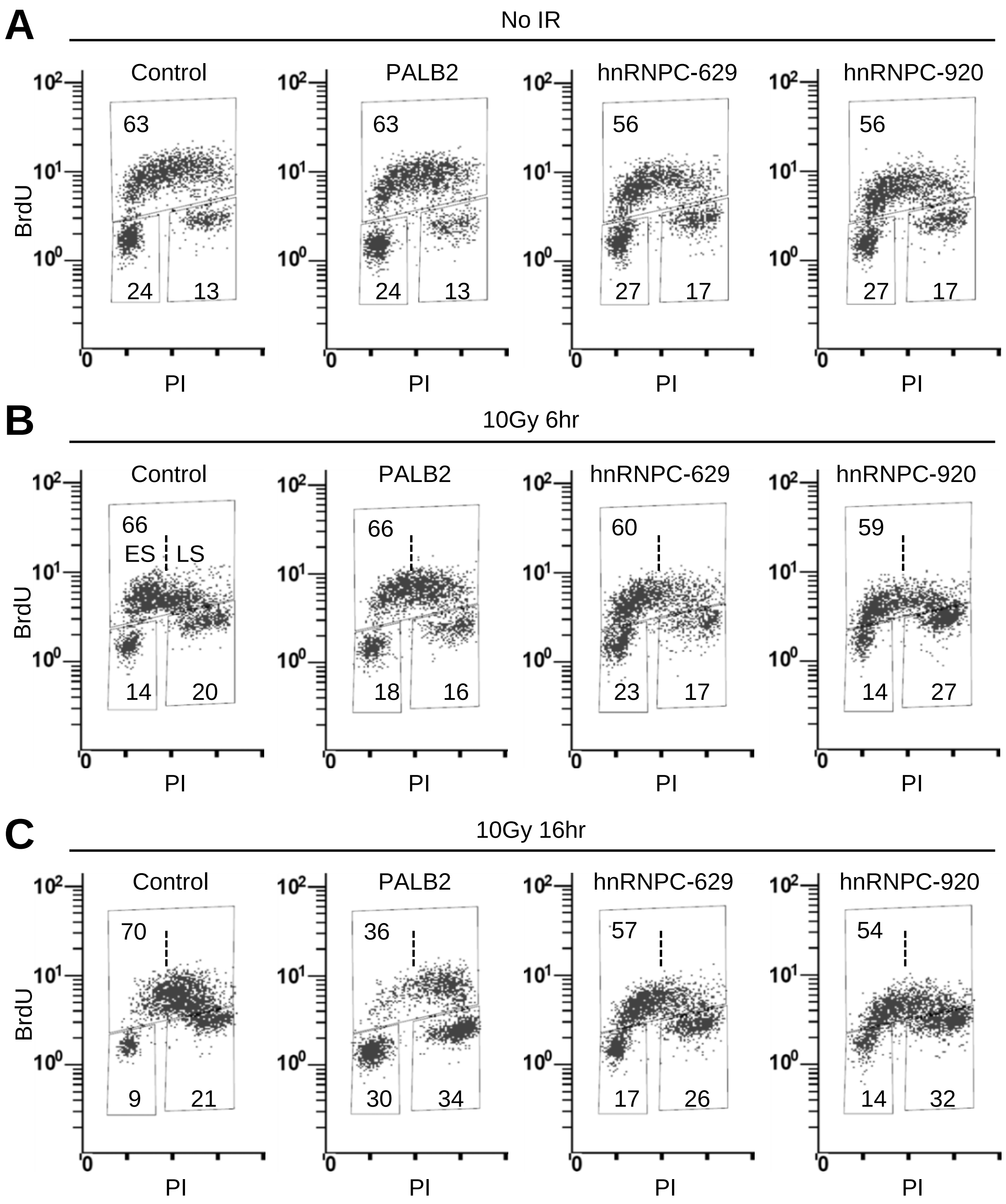

Figure S2. Effect of hnRNP C depletion on cell cycle distribution before and after IR. DR-U2OS cells were treated with control, PALB2 or hnRNP C siRNAs for 72 hr and then subjected to 10 Gy of IR. Cells were labeled with BrdU either before (**A**) or at 6 and 16 hr post IR (**B** and **C**, respectively), and cell cycle profiles were analyzed by anti-BrdU staining and FACS. Cells in S, G1 and G2/M phases were indicated by upper, lower left and lower right boxes, respectively. Numbers in the boxes indicate the percentages of cells in the corresponding phases. In the left panel of B, early S and late S phase cells are indicated by "ES" and "LS" and separated by an arbitrary dotted line.
