# Supplementary material for: Requirement of Heterogeneous Nuclear Ribonucleoprotein C for BRCA Gene Expression and Homologous Recombination
Source: PLoS One. 2013 Apr 9;8(4):e61368. doi: 10.1371/journal.pone.0061368 (PMC3621867; doi:10.1371/journal.pone.0061368)
Supplement: Figure S3 — Comet assay of hnRNP C-depleted cells after IR. A. DR-U2OS cells were treated with control or hnRNP C (1∶1 mix of 629 and 920) siRNAs for 72 hr and then subjected to 10 Gy of IR. Cells were harvested at indicated time points following IR and subjected to alkaline comet assay (Trevigen) following manufacturer's instructions. B. Number (in percentage) of cells with comet tails in a representative experiment. C. Mean length of comet tails in a representative experiment. D–G. Length distribution of comet tails in a representative experiment. Comet measurements were carried out using the Image J software, and approximately 100 comets were measured for each condition. (PDF) [file pone.0061368.s003.pdf]

Figure S3 Anantha et al.

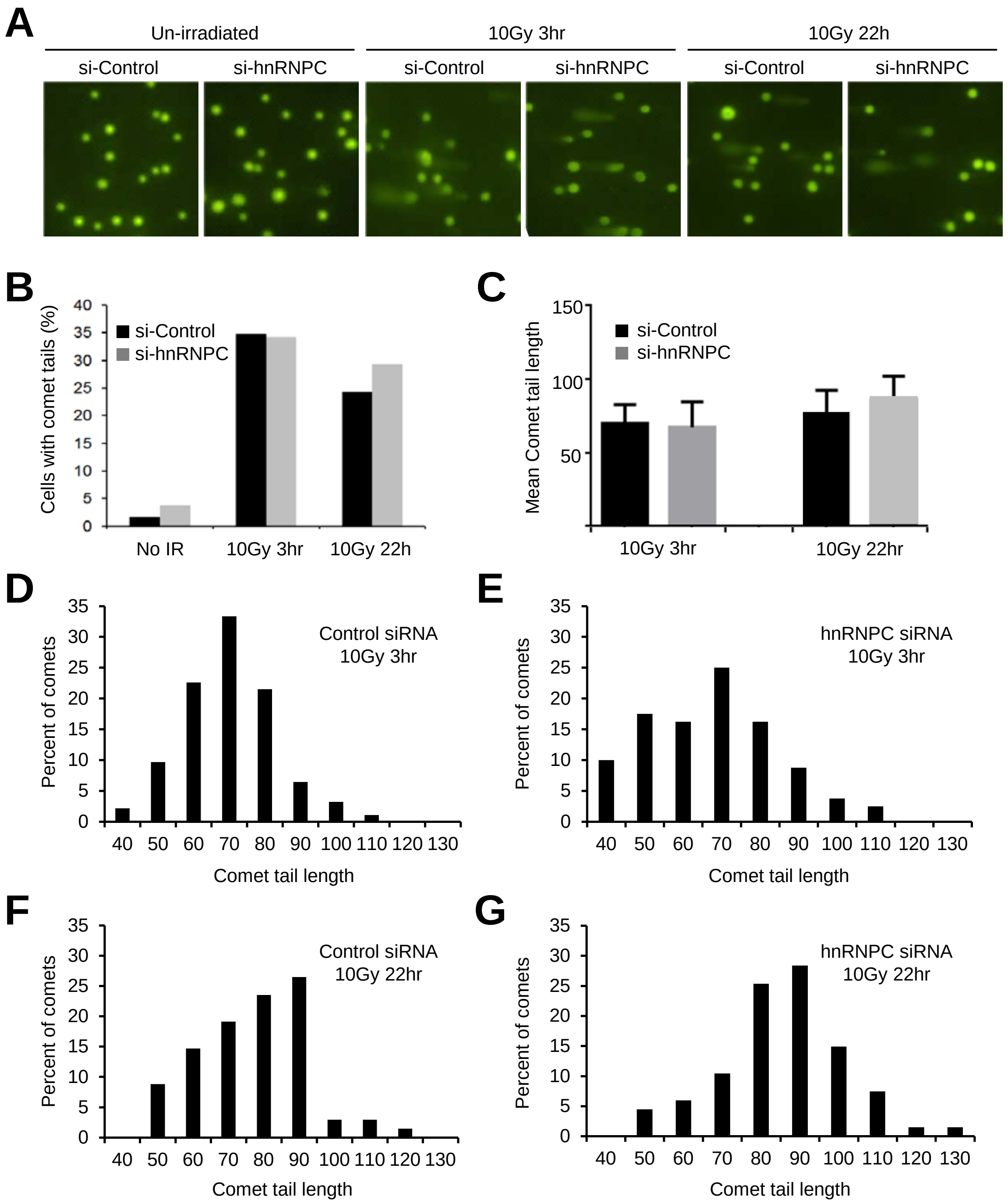

Figure S3. Comet assay of hnRNP C-depleted cells after IR. A. DR-U2OS cells were treated with control or hnRNP C (1:1 mix of 629 and 920) siRNAs for 72 hr and then subjected to 10 Gy of IR. Cells were harvested at indicated time points following IR and subjected to alkaline comet assay (Trevigen) following manufacturer's instructions. **B**. Number (in percentage) of cells with comet tails in a representative experiment. **C**. Mean length of comet tails in a representative experiment. **D**-**G**. Length distribution of comet tails in a representative experiment. Comet measurements were carried out using the Image J software, and approximately 100 comets were measured for each condition.
