# Supplementary material for: Requirement of Heterogeneous Nuclear Ribonucleoprotein C for BRCA Gene Expression and Homologous Recombination
Source: PLoS One. 2013 Apr 9;8(4):e61368. doi: 10.1371/journal.pone.0061368 (PMC3621867; doi:10.1371/journal.pone.0061368)
Supplement: Figure S4 — Reduced abundance and impaired focus formation of BRCA1 and RAD51 in hnRNP C-depleted cells. Control treated or hnRNP C-depleted DR-U2OS cells were subjected to 10 Gy of IR. Cells were fixed at indicated time points and stained for BRCA1 (A) or RAD51 (B) together with γH2A.X. The antibody used were anti-BRCA1 (#07-434, Millipore), anti-RAD51 (sc-8349, Santa Cruz) and anti-γH2A.X (#05-636, Millipore). (PDF) [file pone.0061368.s004.pdf]

Figure S4 Anantha et al.

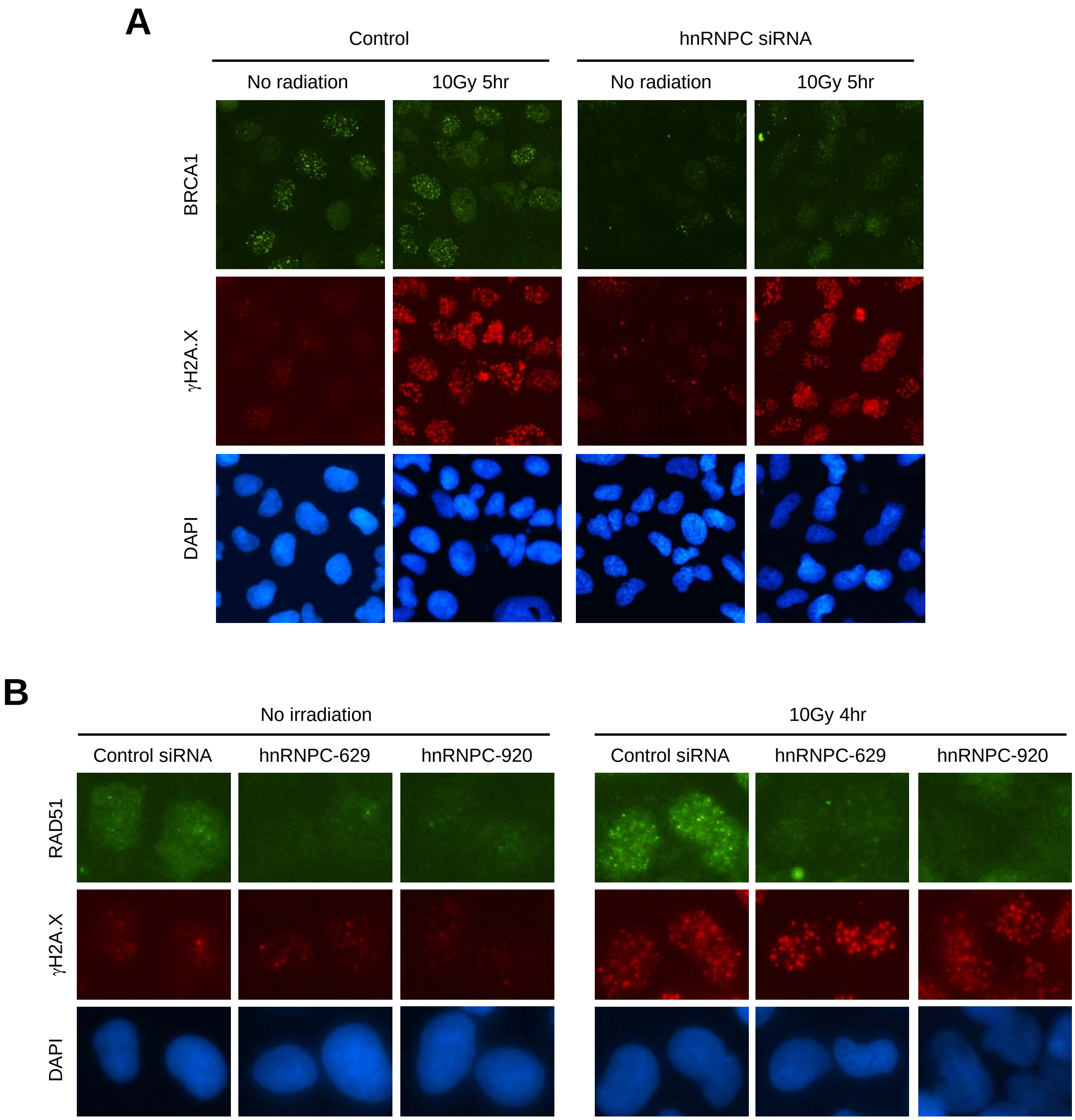

Figure S4. Reduced abundance and impaired focus formation of BRCA1 and RAD51 in hnRNP C-depleted cells. Control treated or hnRNP C-depleted DR-U2OS cells were subjected to 10 Gy of IR. Cells were fixed at indicated time points and stained for BRCA1 (**A**) or RAD51 (**B**) together with γH2A.X. The antibody used were anti-BRCA1 (#07-434, Millipore), anti-RAD51 (sc-8349, Santa Cruz) and anti-γH2A.X (#05-636, Millipore).
