# Supplementary material for: Requirement of Heterogeneous Nuclear Ribonucleoprotein C for BRCA Gene Expression and Homologous Recombination
Source: PLoS One. 2013 Apr 9;8(4):e61368. doi: 10.1371/journal.pone.0061368 (PMC3621867; doi:10.1371/journal.pone.0061368)
Supplement: Figure S5 — Binding of hnRNP C to transcripts of HR genes. A. Genome browser view of PALB2 and BARD1 genes displaying RNA-Seq data (overlapping reads per nucleotide; blue) from control and hnRNP C knockdown HeLa cells, that were independently transfected with two different siRNAs (KD1 and KD2), as well as hnRNP C iCLIP data (crosslink events per nucleotide; purple). RefSeq transcript annotations (blue) and Alu elements in antisense orientation to the shown strand (orange) are depicted below. No Alu exonization events were found in these two genes. B. "Weblogo" showing the base composition at the hnRNP C crosslink sites (position 0) within BRCA1, BRCA2, PALB2, RAD51, BARD1 and BRIP1 gene transcripts as well as the surrounding sequence. The y-axis indicates the informational content for each position in bits. The graph shows the aggregate of all the crosslink sites in the 6 genes. (PDF) [file pone.0061368.s005.pdf]

Figure S5 Anantha et al.

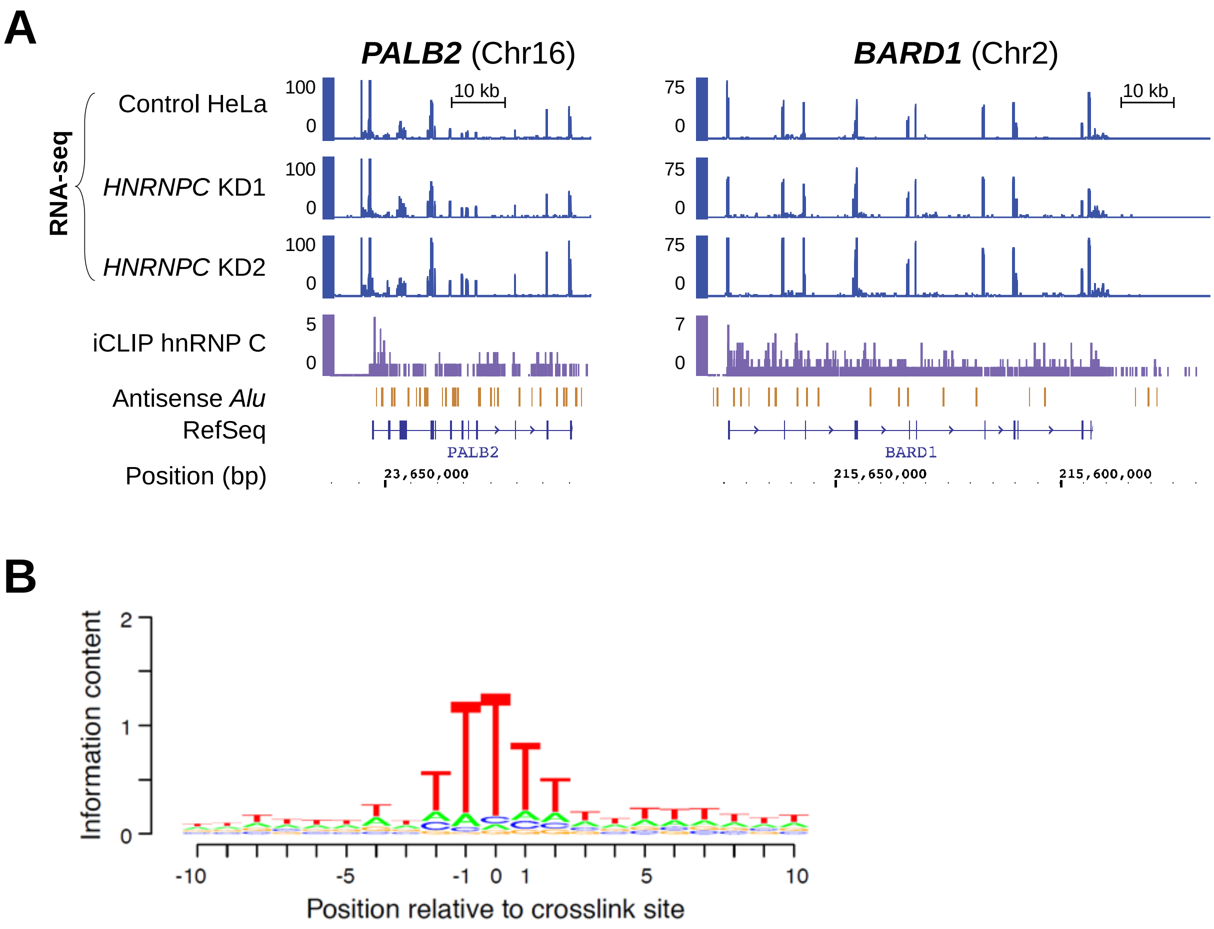

Figure S5. Binding of hnRNP C to transcripts of HR genes. A. Genome browser view of *PALB2* and *BARD1* genes displaying RNA-Seq data (overlapping reads per nucleotide; blue) from control and hnRNP C knockdown HeLa cells, that were independently transfected with two different siRNAs (KD1 and KD2), as well as hnRNP C iCLIP data (crosslink events per nucleotide; purple). RefSeq transcript annotations (blue) and *Alu* elements in antisense orientation to the shown strand (orange) are depicted below. No *Alu* exonization events were found in these two genes. **B.** "Weblogo" showing the base composition at the hnRNP C crosslink sites (position 0) within *BRCA1*, *BRCA2*, *PALB2*, *RAD51*, *BARD1* and *BRIP1* gene transcripts as well as the surrounding sequence. The y-axis indicates the informational content for each position in bits. The graph shows the aggregate of all the crosslink sites in the 6 genes.
